# Post-operative immune suppression is reversible with interferon gamma and independent of IL-6 pathways

**DOI:** 10.1186/2197-425X-3-S1-A820

**Published:** 2015-10-01

**Authors:** ER Longbottom, HDT Torrance, HC Owen, CJ Hinds, RM Pearse, MJ O'Dwyer

**Affiliations:** Barts & the London School of Medicine, QMUL, William Harvey Research Institute, London, United Kingdom; Barts Health NHS Trust, Adult Critical Care Unit, London, United Kingdom

## Introduction

The post-operative period is characterised by increased IL-6 production and clinical features of immune suppression. *In vitro* anti-inflammatory actions of IL-6 are mediated through suppression of interferon gamma (IFNγ) [1]. The clinical significance of IL-6 in mediating post-operative immune suppression remains unclear.

## Objectives

To evaluate the role of IL-6 pathways in post-operative immune suppression and the reversibility of this phenomenon.

## Methods

Patients over 45 years old undergoing elective surgery involving the gastrointestinal tract and requiring at least an overnight hospital stay were recruited. The primary outcome was hospital-acquired infection. IL-6 and IFNγ levels were assayed using ELISA preoperatively and at 24 and 48 hours. Pooled healthy control peripheral blood mononuclear cells (PBMCs) were cultured in perioperative serum and CD14^+^HLA-DR (mHLA-DR) geometric mean florescent intensity (MFI) measured in the presence and absence of interferon gamma (IFNγ) and IL-6 neutralising antibody. Data were analysed with non-parametric statistics.

## Results

**Figure 1:**
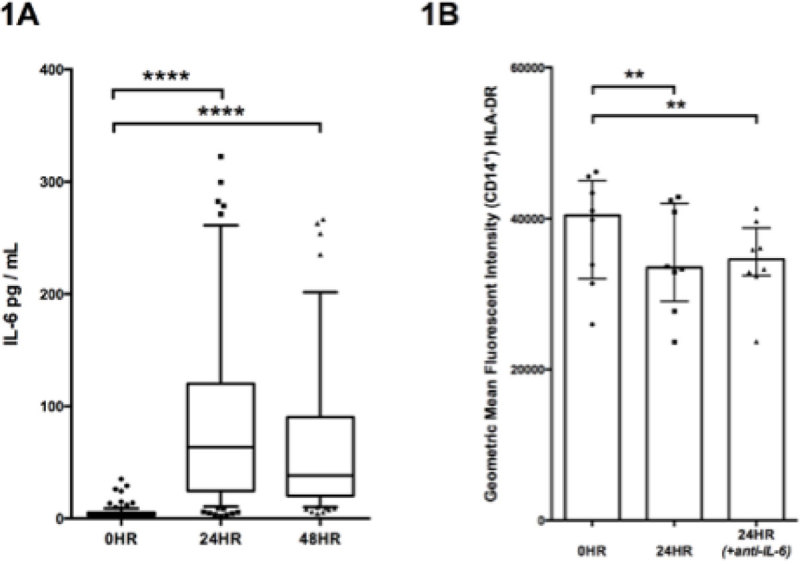
Figure 1

119 patients were recruited and 44 (37%) developed a post-operative infection a median of 9 (IQR 5-11) days postoperatively (Figure 1). IL-6 levels increased from baseline to 24 hours postoperatively (*P* < 0.0001, Figure 1A) but were then unchanged between 24 and 48 hours (*P* = 0.06, Figure 1B). Postoperative IL-6 levels correlated with the duration of the procedure (*P* = 0.009). Higher preoperative IL-6 levels were observed in patients with cancer (*P* = 0.02). IL-6 levels at 24 (*P* = 0.0002) and 48 hours (*P* = 0.003) were associated with the later occurrence of infectious complications. This pattern remained similar after adjustment for baseline characteristics. Healthy donor PBMCs incubated with postoperative serum downregulated mHLA-DR MFI when compared with serum from baseline (n = 8, p = 0.008). Culturing in the presence of IFNγ 250IU (n = 4) prevented this decrease whereas culturing in the presence of IL-6 neutralising antibody 15ng/ml (n = 8) did not.

## Conclusions

IL-6 levels increase following major surgery and are associated with an increased susceptibility to post-operative infections. Serum obtained from post-operative patients induces an immunosuppressive response through an IL-6 independent pathways which is reversible with IFNγ treatment.

## Grant Acknowledgment

The National Institute of Academic Anaesthesia (NIAA)

**Table 1:** Characteristics of patients developing infections and those remaining infection free following scheduled abdominal surgery.

	Infection	Infection free	*P*Value
	*N = 44 (37%)*	*N = 75 (63%)*	
Age (years)	66 (59 - 75)	64 (56 - 71)	0.19
Male sex (%)	27 (61)	47 (63)	0.89
Diabetes (%)	8 (18)	12 (16)	0.76
Current smokers (%)	10 (23)	14 (19)	0.60
Cancer diagnosis (%)	24 (55)	53 (71)	0.07
Preoperative Immunosuppression (%)	6 (14)	10 (14)	>0.99
Duration of operation (minutes)	243 (176 - 312)	195 (142 - 295)	0.06
Data are described as median with interquartile range with percentages in parenthesis			
